# The anti-melanoma activity and oncogenic targets of hsa-miR-15a-5p

**Published:** 2016-11-14

**Authors:** Christopher Alderman, Yixin Yang

**Affiliations:** Department of Biological Sciences, Emporia State University, 1 Kellogg Circle, Emporia, Kansas, 66801 USA

**Keywords:** rmcroRNA-15a, cancer, CDCA4, BCL2L2, YAP1, AKT-3, CCNE1, Cyclin El, γ-Synuclem

## Abstract

MiRNAs regulate gene expression post-transcriptionally and pre-translationally. Through gene regulation, several miRNAs have been found to play a significant role in various diseases. Each miRNA has multiple targets and is able to have a potent, albeit complex, effect on the cells. Specifically, miRNA-15a has been found to significantly reduce cancer cell survival and aggressiveness through multiple mechanisms across several cancer types. Our research found that miRNA-15a was able to decrease melanoma cell viability in-vitro and in-vivo. We have also found that miRNA-15a caused cell cycle arrest at the G_0_/G_1_ phase. Moreover, miRNA-15a was found to decrease the invasiveness of melanoma cells. CDCA4 was also discovered as a novel *bona-fide* target of miRNA-15a. The following oncogenic mRNAs are verified targets of miRNA-15a: CDCA4, BCL2L2, YAP1, AKT-3, Cyclin E1, and γ-Synuclein. In the future we hope to better understand which miRNAs will be effective in different transcriptome and genome environments. Efforts such as the NIH Center for Cancer Genomics' ‘The Cancer Genome Atlas,’ ‘Cancer Target and Driver Discovery Network,’ and the ‘Human Cancer Models Initiatives’ among others, will help us characterize the specific tumor environments in which different miRNAs are able to reduce cancer proliferation and aggression. This information will be enhanced by improving the delivery of miRNA by inducing its expression in-situ with dCas9 conjugated to activation domains.

MicroRNA (miRNA, miR) is a 22 nucleotide non-coding RNA molecule that regulates translation by binding the RNA-Induced Silencing Complex (RISC) with Ago 1-2 (E1f2C1-2), and Gemin3-4 ^[1-3]^. The RISC-miRNA complex binds mRNA and inhibits its translation through a variety of methods. If the complementary strands of miRNA and mRNA are near-identical, nuclease activity is observed by Argonaute on the mRNA strand in cytoplasmic “P-bodies” ^[4, 5]^. This leads to complete degradation of the mRNA and inability for it to express its protein. However, even partial complementary matches are able to inhibit translation by blocking ribosome binding ^[4]^.

Due to its ability to control cell processes by modulating protein expression, miRNA has been studied heavily in order to observe its ability to fight disease. Recently, many miRNAs have shown promise in being able to modulate the behaviors of cancer cells through regulating various target mRNAs ^[6]^. In fact, there are currently at least five miRNAs or sister-like siRNAs in clinical trials for cancer ^[7]^. It is crucial for us to understand the complex interactions of miRNAs, transcription factors, mRNAs, and effector proteins. By clarifying these interactions, researchers will be able to guide the future of miRNA research and clinical applications.

Different cancer types tend to have characteristic miRNA deregulation profiles that can be used to make specific treatment plans for individual patients ^[8]^. Several deregulated miRNAs have been established in melanoma cells such as miR-26a and miR-15a ^[9, 10]^. In breast cancer, the miR-200 family has shown regulation of tumor progression and miR-205 inhibits metastasis ^[11, 12]^. Moreover, colorectal cancer metastasis and patient survival have been shown to be regulated by miR-184 and miR-133b ^[13]^. Several types of cancers are also affected by deregulation of miRNAs that control DNA-repair mechanisms ^[14]^. For example, miR-181d targets MGMT, which is crucial for DNA repair and several miRNAs target DNA repair enzyme inhibitor HGMA2 ^[15, 16]^. These are some of the best known miRNA deregulations in cancer, but there are more being discovered and validated. Not only can we treat these cancer patients with miRNA, but we are also able to use miRNA as an oncogenic biomarker early detection method and for understanding the mutation spectrum of certain cancer populations ^[17]^.

MiRNA-15a-5p has shown multiple strong roles in regulating cancer cells across several cell types. While there have been many theoretical targets of miRNAs, it is important to make sure they have been properly validated before making assumptions on their activity from computations alone. With this in mind, we have used only evidence-based knowledge to help clarify what is currently known about miR-15a activity in cancer. There are several pathways that interact together through miR-15a. In fact, miR-15a has activity on multiple transcription factors, making its role in cancer quite complex. Together, all of the effects of miR-15a lead to anti-cancer effects from multiple directions, making it an ideal cancer therapy molecule.

For the US alone, there are an estimated 595,690 deaths caused by cancer over the year of 2016 ^[18]^. In the US, melanoma is forecasted to consist of 6% of all new cancer cases for males and 3% for females as well as result in death for 10,130 cancer patients. Effective and safe treatment options are still in high demand. One of the most effective targeted treatments for melanoma, Vemurafenib in combination with Cobimetinib, still only has median progression free survival of 9.9 months and 24% of patients obtain Cutaneous Squamous Cell Carcinoma as a side-effect ^[19, 20]^. Moreover, although interleukin-2 and Ipilimumab have recently been credited for their relatively high efficacy against melanoma, they still only have a 5-year survival rate of 20% ^[21, 22]^. The need for new therapies is prevalent, and miRNA is starting to fill that role.

We have been working to discover the effects of miR-15a in malignant melanoma. We have found that miR-15a displayed a strong inhibitory effect on cell proliferation, cell cycle progression and cell migration of melanoma cells and directly targeted CDCA4 ^[10]^. We observed that miR-15a transfection significantly decreased the cell viability of four different melanoma cell lines: B16-F10, SKMEL-28, A375 and CRL-2808. Also, miR-15a caused increased percentages in the G_1_/G_0_ phase and concomitant decrease in cell populations in both the S and G_2_ phases, suggesting that miR-15a caused cell cycle arrest at G_1_/G_0_ phase. In addition, miR-15a transfection reduced the cell invasion by 47%, which was displayed by the decreased ability of the melanoma cells to invade through the Transwell membrane. *In vivo*, miR-15a significantly retarded the growth of melanoma established by melanoma cells that were transfected with miR-15a starting at 10 nM. We also performed a mechanistic study to discover the mechanism by which miR-15a induces these anti-cancer properties. We used the TargetScan database to determine highly conserved theoretical targets of miR-15a. Putative targets were then analyzed through Western Blotting to determine whether these targets were down-regulated by miR-15a. We found Akt-3 and CDCA4 to both have decreased expressions in both the CRL-2808 and SK-MEL-28 cell lines. In order to determine the *bona-fide* target of miR-15a, we used a luminescence reporter assay, in which a Gaussia Luciferase (GLuc) gene was under the regulation of CDCA4's 3′ untranslated region (UTR) on its 3′ end. While the scramble miRNA had no effect on GLuc expression, miR-15a mimics successfully inhibited the expression and activity of luciferase. Since miR-15a significantly decreased expression of the CDCA4 3′ UTR-regulated gene, we conclude that miR-15a directly targets the CDCA4 3′ UTR at the 468-475 highly conserved seed region as determined by TargetScan. This experiment was the first elucidation of CDCA4 as a direct target of miR-15a. CDCA4 activity has been found to be associated with the G1/S phase transition regulation as a transcription factor. It is a Trip-Br cofactor, which is known to suppress the E2F1 promoter ^[23]^. CDCA4 has also been determined to be a transcription factor that plays a role in regulating the expression of the JUN oncogene ^[24]^. Our lab is currently working on elucidating more functions and associations with the CDCA4 protein.

One of the first studies done to understand the anti-cancer effects of miR-15a was in 2009 where AKT-3 was determined as a novel direct target. In 2003 Stahl *et al*. determined that AKT-3 deregulation drove melanoma cell proliferation ^[25]^. Moreover, in 2008 Cheung *et al*. discovered that AKT-3 works synergistically with V600E Braf to exacerbate melanoma cell proliferation ^[26]^. A 2009 study by Roccaro *et al*. then elucidated AKT-3 as a target of miR-15a in multiple myeloma ^[27]^. Another discovery in 2013 by Luo *et al*. showed that miR-15a is able to inhibit breast cancer by targeting cyclin E1 (CCNE1) ^[28]^. Cyclin E1 is able to regulate the G1 to S phase transition in the cell cycle by regulating the activity of CDK2, a protein whose activity is mandatory for progressing from the G1 to S phase ^[29]^. This function of CDK2 has been known to be tumorigenic and makes cyclin E1 a desirable target for cancer treatment ^[30]^.

A 2015 study by Kang *et al*. showed that miR-15a displays anti-cancer activity by targeting Yes-associated protein 1 (YAP1) ^[31]^. These results were determined through multiple experiments with gastric adenocarcinoma (GAC). Kang *et al*. found miR-15a to be downregulated in GAC cells and to have an inverse expression relationship with YAP1. YAP1 is a transcription factor that has several characteristics that make it a target of interest in cancer biology. It has been shown that YAP1 plays an integral role in tumor cell survival by rescuing K-Ras, B-Raf, and PI3K inhibition ^[32]^. This makes it an important protein to target as an adjuvant therapy with K-Ras, B-Raf, and PI3K inhibitors in cancer treatment. YAP1 also has been identified to antagonize contact inhibition when dephosphorylated by activity initiated through mitogenic growth factors, allowing nuclear localization and induction of CTGF expression ^[33]^.

In 2015 it was discovered that BCL2L2 is also targeted by miR-15a in non-small cell lung cancer ^[34]^. This was further supported by results in 2016 that confirmed BCL2L2 as a target of miR-15a in HPV-positive hypopharyngeal squamous cell carcinoma ^[35]^. BCL2L2 protein is in the BCL2 family and works as an anti-apoptotic molecule by preventing BH123 proteins from releasing cytochrome C from the mitochondria. These experiments were further proved by observing a reverse effect on cancer proliferation after the use of miR-15a inhibitors. In addition to targeting BCL2L2, miR-15a is able to induce apoptosis in some breast cancer cells by targeting γ-Synuclein (SNCG) ^[36]^. γ-Synuclein is known to be correlated with poor prognosis in breast cancer, colon adenocarcinoma, oral squamous cell carcinoma, and bladder cancer ^[37-40]^. The gene targets of miR-15a identified up to date and the effects of downregulating these target genes are demonstrated in Figure 1.

MiRNA is very effective and its abilities are far-reaching, with the ability to regulate up to 80% of the human genome ^[41]^. In future studies, it is becoming more important that we find ways to use our information about the cancer microRNAome in translational research to determine effective and affordable clinical treatment plans. Much is known about the cancer microRNAome, but until we have a dependable way to treat cancer patients with miRNA, this knowledge is not reaching its full potential ^[6]^. Currently, clinical trials are underway for several microRNAs in what is called “miRNA replacement therapy.” MiRNA mimics and expression vectors are used for the main method of delivery in this kind of therapy ^[42]^. Unfortunately, miRNA mimics are expensive, have a short half-life *in vivo*, and have poor biodistribution; moreover, even though use of expression vectors is efficient, it has high toxicity and also creates immunogenic problems ^[43]^. Fortunately, recent researchers have discovered more about how miRNA is transferred between cells. Using this information alongside the use of CRISPR interference (CRISPRi) to control the expression of miRNA, we may be able to provide cancer populations with translation control that will help regulate the cell cycle and decrease the malignancy of cancer populations.

Our lab is currently working on an innovative technique that employs a variation of CRISPR/Cas9 to specifically target the promoter region of the miR-15a gene and other microRNA genes. CRISPR/Cas9 works as revolutionary genome-editing tool by synthesizing crRNA to target any specific position of the genome with relatively high fidelity ^[44]^. This technique can be used to modify the genome or target the genome with transcription factors directly. CRISPRi is performed by conjugating transcription activator domains to a ‘dead’ (endonuclease-inactivated) version of Cas9, dCas9, thus enabling one to induce transcription of almost any gene, including miRNAs, in the genome ^[45]^. The CRISPR system is quite affordable and costs only about 50 USD per guide RNA and necessary components; old methods for genome targeting such as the zinc finger nucleases and TALENS required thousands of dollars and the laborious task of making custom proteins rather than guide RNAs ^[46, 47]^.

The CRISPR/Cas9 system by itself may not be the best method of action for fighting cancer mutations, though, because if even one cell is not successfully edited, the entire cancer population could be regenerated. On the other hand, with the new research done on understanding circulating microvesicles (CMVs), budded off cell fragments ranging from 100-1000 nm in diameter, miRNA is found to be passed between cells on a large scale ^[48-51]^. Therefore, by inducing the transcription of anticancer miRNA in any significant number of cells, cancerous or not, all the cancerous cells will be saturated with the necessary translation-regulating miRNAs. In fact, clinical trials are in progress for using these microvesicles in the early detection of cancers; although, to date, there have been no other studies working with the cancer microRNAome on its combined use with CRISPR/Cas9 for the treatment of cancer cells ^[17, 52]^.

The novel idea of targeting the regulation of miRNA with CRISPRi presented here is hopeful because it provides a method of sustained regulation of gene expression across the entire cancer population so that small sub-populations are not able to regenerate the cancer cell population. As previously mentioned, this method of miRNA delivery will improve cost, efficacy, treatment frequency, miRNA stability, toxicity, biodistribution, and immunogenic problems. In order for this to work clinically, significant amounts of research will need to be done to improve the fidelity of CRISPR/Cas9 and observe the efficacy of this method in the lab under various circumstances ^[53]^. It is our hope that significant progress will be made on this in the scientific and public community so that reliable, safe treatments for cancer patients can be provided at the soonest time possible.

## Figures and Tables

**Figure 1:**
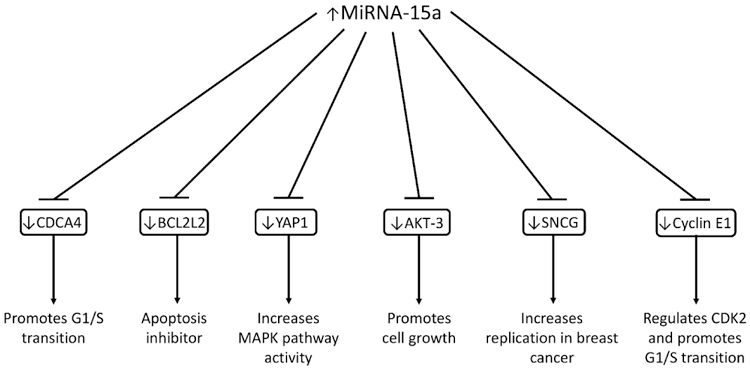
The complete gene targets of miR-15a identified to date and the effects of downregulating these target genes.
